# Genome-Wide and Gene-Specific Epigenomic Platforms for Hepatocellular Carcinoma Biomarker Development Trials

**DOI:** 10.1155/2014/597164

**Published:** 2014-04-17

**Authors:** Christina Michailidi, Ethan Soudry, Mariana Brait, Leonel Maldonado, Andrew Jaffe, Carmen Ili-Gangas, Priscilla Brebi-Mieville, Jimena Perez, Myoung Sook Kim, Xiaoli Zhong, Quiang Yang, Blanca Valle, Stephen J. Meltzer, Michael Torbenson, Manel Esteller, David Sidransky, Rafael Guerrero-Preston

**Affiliations:** ^1^Department of Otolaryngology, Division of Head and Neck Cancer Research, School of Medicine, The Johns Hopkins University, Baltimore, MD 21231, USA; ^2^Department of Epidemiology, Bloomberg School of Public Health, The Johns Hopkins University, Baltimore, MD 21205, USA; ^3^Laboratorio de Patología Molecular, Departamento de Anatomí a Patológica, CEGIN-BIOREN, Facultad de Medicina, Universidad de La Frontera, 4781176 Temuco, Chile; ^4^Department of Gastroenterology, School of Medicine, The Johns Hopkins University, Baltimore, MD 21205, USA; ^5^Department of Pathology, School of Medicine, The Johns Hopkins University, Baltimore, MD 21205, USA; ^6^Bellvitge Biomedical Research Institute, Barcelona, Catalonia, Spain; ^7^Department of Obstetrics and Gynecology, University of Puerto Rico School of Medicine, San Juan, PR 00927, USA

## Abstract

The majority of the epigenomic reports in hepatocellular carcinoma have focused on identifying novel differentially methylated drivers or passengers of the oncogenic process. Few reports have considered the technologies in place for clinical translation of newly identified biomarkers. The aim of this study was to identify epigenomic technologies that need only a small number of samples to discriminate HCC from non-HCC tissue, a basic requirement for biomarker development trials. To assess that potential, we used quantitative Methylation Specific PCR, oligonucleotide tiling arrays, and Methylation BeadChip assays. Concurrent global DNA hypomethylation, gene-specific hypermethylation, and chromatin alterations were observed as a hallmark of HCC. A global loss of promoter methylation was observed in HCC with the Illumina BeadChip assays and the Nimblegen oligonucleotide arrays. HCC samples had lower median methylation peak scores and a reduced number of significant promoter-wide methylated probes. Promoter hypermethylation of * RASSF1A*, * SSBP2*, and * B4GALT1* quantified by qMSP had a sensitivity ranging from 38% to 52%, a specificity of 100%, and an AUC from 0.58 to 0.75. A panel combining these genes with HCC risk factors had a sensitivity of 87%, a specificity of 100%, and an AUC of 0.91.

## 1. Introduction

Promoter-wide alterations of DNA methylation have been described at all stages that encompass hepatocarcinogenesis, precancerous lesions, and tumor initiation to unresectable HCC [1, 2], mostly focusing on aberrant hypermethylation of CpG islands in gene promoter regions near the Transcription Start Site (TSS) [3, 4]. Several hypermethylated genes have been identified using a range of diverse technologies. On the other hand, studies are also reporting promoter hypomethylation in specific genes to play an important role in HCC [5, 6], suggesting that high frequencies of hypomethylation, in various cancers, would be valuable as a cancer diagnostic marker.

The earlier methylation studies of HCC used the candidate gene approach and first generation methylation microarrays, which study less than 7K CpG islands [7]. A recent number of studies have used Methylated DNA immunoprecipitation-on-chip analysis (MeDIP-chip) [8] and BeadChip assay technologies to identify novel genes differentially methylated in HCC [9]. Most HCC methylation studies have used available technologies to profile and identify differentially methylated genes that drive the oncogenic process [10].

The contribution of DNA methylation to the development of HCC is not yet elucidated. A methylation study in HCC is also challenging as there are several well-known risk factors for HCC, such as alcohol-induced cirrhosis and chronic viral hepatitis B or C infection [11]. Aberrant DNA methylation profiles across the genome were identified in tumor tissues from US HCC cases that are predominantly related to HCV infection [12]. Yet not much emphasis has been placed on using existing methylation platforms to evaluate differentially methylated genes as biomarkers of HCC, regardless of whether these alterations are driving the oncogenic process or are molecular changes that occur during malignant transformation.

We selected two existing methylation platforms to separately distinguish between HCC and non-HCC liver tissue in a small number of samples: an oligonucleotide methylation tiling array (MeDIP-chip, Nimblegen's 385K Promoter, and CpG Island methylation array) and the Infinium Human Methylation 27K BeadChip assay (Illumina). We then generated a list of hypermethylated genes based on both the frequency in which the genes had been identified as methylated in different studies and also in the methylation arrays we used. From this list, we chose three genes for validation in an independent cohort comprised of HCC and adjacent nonpathological samples using quantitative Methylation-Specific PCR (qMSP). The focus of the study was to identify whether methylation platforms stratifying a small sample size together with publicly available genomic and epigenomic databases could be deployed in biomarker development trials. The methylation platforms can be used as stand-alone tools or as complementary platforms to other transomic tools, depending on the scientific question.

## 2. Materials and Methods

### 2.1. Patient Selection

Deidentified frozen primary HCC, adjacent nontumor (cirrhotic and noncirrhotic), and normal liver (noncirrhotic tissue obtained from autopsies) tissue samples were obtained from the Johns Hopkins University School of Medicine and the Human Cooperative Tissue Network. The study protocol conforms to the ethical guidelines of the 1975 Declaration of Helsinki as reflected in a priori approval by the Johns Hopkins Institutional Review Board. All patients had not undergone therapy prior to sample collection. The samples were frozen in liquid nitrogen and stored at −80°C.

### 2.2. DNA Extraction, Bisulfite Conversion, and MeDIP Enrichment

Tissue samples were digested with 1% SDS and 50 *μ*g/mL proteinase K (Bushranger Mannheim) at 48°C overnight, followed by phenol/chloroform extraction and ethanol precipitation of DNA as previously described [13]. Prior to using qMSP and the Illumina BeadChip assay, bisulfite modification of 2 *μ*g of genomic DNA was performed as previously described [14]. Prior to using the Nimblegen tiling arrays, 500 ng of genomic DNA was sheared using a water bath sonicator (Bioruptor UCD-200, Diagenode). Sonicated DNA was then analyzed on a 1.5% agarose gel to ensure that it had an optimal size of 200–1000 bp. MeDIP was subsequently performed with the Methyl DNA Immunoprecipitation Kit (Epigentek). Fractions of Input DNA and Immunoprecipitated DNA from each sample were subsequently sent to Nimblegen for labeling, hybridization, and scanning.

### 2.3. Illumina BeadChip Array

Bisulfite-treated DNA from 3 HCC samples and 3 adjacent normal liver samples was hybridized to the Human Methylation 27K BeadChip, which quantitatively interrogates 27,578 CpG loci covering more than 14,000 genes at single-nucleotide resolution. The Infinium Methylation assay detects cytosine methylation at CpG islands based on highly multiplexed genotyping of bisulfite-converted genomic DNA (gDNA). The assay interrogates these chemically differentiated loci using two site-specific probes, one designed for the methylated locus (M bead type) and another for the unmethylated locus (U bead type). Single-base extension of the probes incorporates a labeled ddNTP, which is subsequently stained with a fluorescence reagent. The level of methylation for the interrogated locus can be determined by calculating the ratio of the fluorescent signals from the methylated versus unmethylated sites.

### 2.4. Nimblegen 385K CpG Island Plus Promoter Array

DNA (500 ng) from 3 liver tissue samples (1 HCC and 2 noncirrhotic normal liver samples) enriched with MeDIP were hybridized to Nimblegen Promoter plus CpG Island 385K oligonucleotide tiling arrays. A single array design covers 28,226 CpG islands and promoter regions for 17,000 RefSeq genes. The promoter region covered is 1 kb long: 800 bp upstream from the TSS and 200 bp downstream from the TSS. Small CpG islands are extended at both ends for a total additional coverage of 700 bp for more reliable detection. DNA methylation positive control regions, such as the HoxA gene cluster, H19/IGF2 cluster, KCNQ1 cluster, and IGF2R gene, are also included on the array.

### 2.5. Spotfire Analysis and Heat Map Creation

The beta values of all probes on the Illumina Infinium arrays were subjected to log10 transformation in order to generate a dendrogram and corresponding heat map based on unsupervised hierarchical clustering with Spotfire (Somerville, MA). The clustering was performed with the unweighted average method using correlation as the similarity measure and ordering by average values.

### 2.6. Bioinformatics Analysis of Methylation Array Data

The Microarray Core at Johns Hopkins School of Medicine performed the bioinformatics analysis of the Infinium array data using Illumina's proprietary BeadStudio software package to provide average methylation Beta values for each probe. Nimblegen performed the bioinformatics analysis for the 385K CpG Island Plus Promoter Array. Nimblegen uses the ACME algorithm to identify hypermethylated genes that have a statistically significant methylation peak score above 2 [15].

### 2.7. Gene Selection from Public Databases of Known Methylation Events in Cancer

Candidate gene selection for promoter methylation analysis was performed utilizing existing databases of known methylation events in cancer [16, 17]. We generated a list of hypermethylated genes based on both the frequency in which the genes had been identified as methylated in different studies and also in the methylation arrays we used. From this list, we chose three genes for validation in an independent cohort comprised of HCC and adjacent nonpathological samples using quantitative Methylation-Specific PCR (qMSP). qMSP primers and probes span an 800 bp region upstream from the TSS. The genes chosen were sequence-specific single-stranded DNA-binding protein 2 (*SSBP2*), which has been previously shown to be hypermethylated in other solid tumors [18], beta-1,4-galactosyltransferase-1 (*B4GALT1*), not previously shown to be hypermethylated in cancer, and the Ras association domain family member 1 (*RASSF1A*), already shown to be hypermethylated in HCC [10].

### 2.8. Quantitative Methylation-Specific PCR

DNA from 27 HCC and 22 adjacent normal tissue samples (cirrhotic, noncirrhotic, and cryptogenic) was bisulfite treated and analyzed with qMSP. Fluorogenic PCR reactions were carried out in a reaction volume of 20 *μ*L consisting of 600 nmol/L of each primer, 200 *μ*mol/L probe, 0.75 units platinum Taq polymerase (Invitrogen), 200 *μ*mol/L of each dATP, dCTP, dGTP, and dTTP, 200 nmol/L ROX dye reference (Invitrogen), 16.6 mmol/L ammonium sulfate, 67 mmol/L Trizma (Sigma, St. Louis, MO), 6.7 mmol/L magnesium chloride, 10 mmol/L mercaptoethanol, and 0.1% DMSO. Duplicates of three microliters (3 *μ*L) of bisulfite-modified DNA solution were used in each real-time methylation-specific PCR (qMSP) amplification reaction. Primers and probes were designed to specifically amplify the promoters of the three genes of interest (*RASSF1A*,* SSBP2*, and* B4GALT1*) and the promoter of a reference gene, actin-B (*ACTB*). Primer and probe sequences and annealing temperatures are provided in Supplementary Table 1 in Supplementary Material available online at http://dx.doi.org/10.1155/2014/597164.

Amplification reactions were carried out in 384-well plates in a 7900 HT Fast Real-Time PCR System (Applied Biosystems) and were analyzed by SDS 2.2.1 Sequence Detector System (Applied Biosystems). Thermal cycling was initiated with a first denaturation step at 95°C for 3 minutes, followed by 40 cycles of 95°C for 15 seconds, and 58°C for 1 minute. Each plate included patient DNA samples, positive (Bisulfite-converted Universal Methylated Human DNA Standard, Zymo Research) and negative (normal leukocyte DNA or DNA from a known unmethylated cell line) controls, and multiple water blanks. Serial dilutions (60 ng, 6 ng, 0.6 ng, 0.06 ng, and 0.006 ng) of Bisulfite-converted Universal Methylated Human DNA Standard were used to construct a standard curve for each gene.

### 2.9. Statistical Analysis for qMSP and Methylation Array Data

qMSP values were adjusted for DNA input by expressing results as ratios between 2 absolute measurements. The relative level of methylated DNA for each gene in each sample was determined as a ratio of qMSP for the amplified gene to* ACTB* and then multiplied by 100 for easier tabulation ((average DNA quantity of methylated gene of interest/average DNA quantity for internal reference gene b-actin) × 100) [19]. The samples were categorized as unmethylated or methylated based on detection of methylation above a threshold set for each gene. For quality control, all amplification curves were visualized and scored without knowledge of the clinical data. ROC curves were used to identify a cut-off ratio above the highest control ratio observed for each gene to set specificity at 100% [10]. Hypermethylation ratios for each gene were compared between cancer HCC and non-HCC samples. Once the best individually discriminating genes were found, 2-gene and 3-gene panels were tested to identify the highest sensitivity with specificity set at 100% for each gene.

## 3. Results

Patient characteristics are summarized in Table 1 (note that, for some samples analyzed by qMSP, clinical information was missing and only patients' histology was known). The majority (58%) of the patients in our study were men. The mean age of the patients was 47.3 years, and most (56%) of the patients were over 50 years old. The ethnicities of the patients in our study were White (74%), Black (23%), and Asian (2%). The most frequent HCC risk factor seen in the patients of this study was viral infection with HCV (35%) or HBV (5%). Interestingly, cryptogenic cirrhosis was seen in 26% of the patients. Alcohol intake was the risk factor for a handful of patients (5%). “M” represents that a sample has a value above the qMSP methylation threshold for that gene. This sample is methylated. “U” represent that a sample has a value below the qMSP methylation threshold for that gene. This sample is not methylated

### 3.1. Global and Gene-Specific Differential DNA Promoter Methylation Arrays

**Figure 1:**
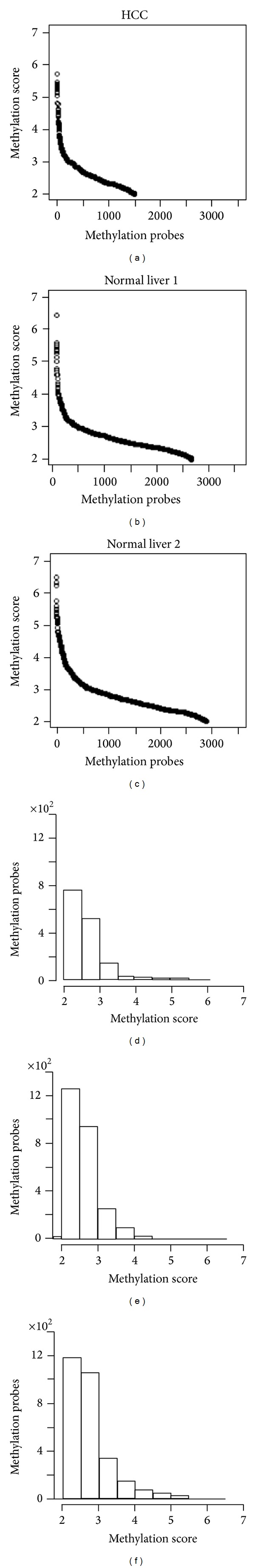
Scatterplots and histograms for a representative set of one tumor sample and two normal samples hybridized to oligonucleotide methylation tiling arrays. The methylation score is on the *Y*-axis of the scatterplots and the number of methylated probes is on the *X*-axis. The number of methylated probes is on the *Y*-axis of the histograms and the methylation score is on the *X*-axis.

We used scatterplots to compare differential DNA methylation values between HCC and normal liver tissue samples hybridized to the 385K Nimblegen tiling array after DNA enrichment with MeDIP (MeDIP-chip).  Figure 1 shows representative scatterplots and histograms in which a decrease in global DNA promoter methylation clearly distinguishes between HCC and normal tissue. Scatterplots and histograms of genome-wide DNA methylation array data provide a snapshot of the differences in methylation patterns between tumor and normal samples. Genome-wide hypomethylation was observed in the tumors when compared to normal samples. A representative tumor sample has less significant methylated probes (1,503) than either one of the normal liver tissue samples (2,585 and 2,887, resp.). Furthermore, the median methylation score was significantly lower for the tumor sample (5.7) than for the normal samples (6.7).

**Figure 2:**
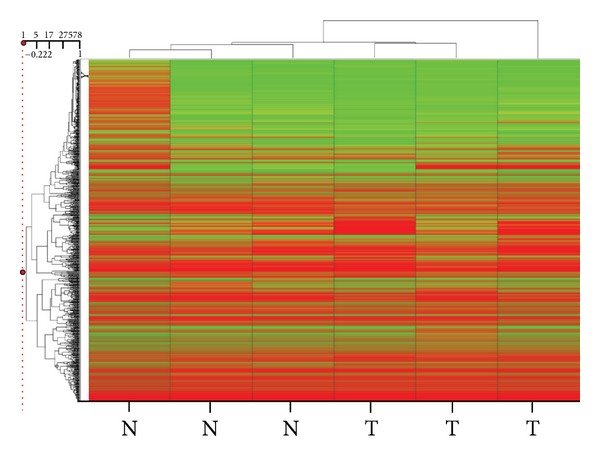
Heat map of the promoter-wide methylation data obtained by hybridizing to the Infinium array three hepatocellular carcinoma (HCC) samples and three nontumor liver samples from patients with no known liver disease. A dendrogram (tree graph) of the average beta values for three HCC samples and three nontumor samples was created with Spotfire (Somerville, MA). Unsupervised hierarchical clustering was performed with the unweighted average method using correlation as the similarity measure and ordering by average values. The color red was selected to represent high scores and the color green to represent low scores.

We used unsupervised clustering of the Illumina BeadChip array results to create a heat map based on correlation, which clearly separates the three HCC samples from the adjacent normal liver sample (Figure 2). Please note that, for one normal sample, even though it clustered with the other two adjacent normal samples, probe-specific methylation levels were higher than expected.

### 3.2. Promoter Hypermethylation in Tumor and Adjacent Normal Samples

Our search of publicly available methylation databases found a combined total of 549 methylated genes when searching for hepatocellular carcinoma (389) and hepatoma (160), 451 of which were unique genes. After crossing that list with the list of frequently methylated genes we identified using methylation arrays, we chose three genes for validation, one gene that was already found to be hypermethylated in HCC by several groups (*RASSF1A*) and two genes that we have reported as methylated in other tumors but not in HCC (*B4GALT1* and* SSBP2*). We quantified the promoter methylation of these 3 genes in 27 HCC samples and 22 adjacent normal samples. To determine the frequency of methylation, we used primers and probes for qMSP previously designed in our laboratory based on bisulfite sequencing data [20, 21].

**Figure 3:**
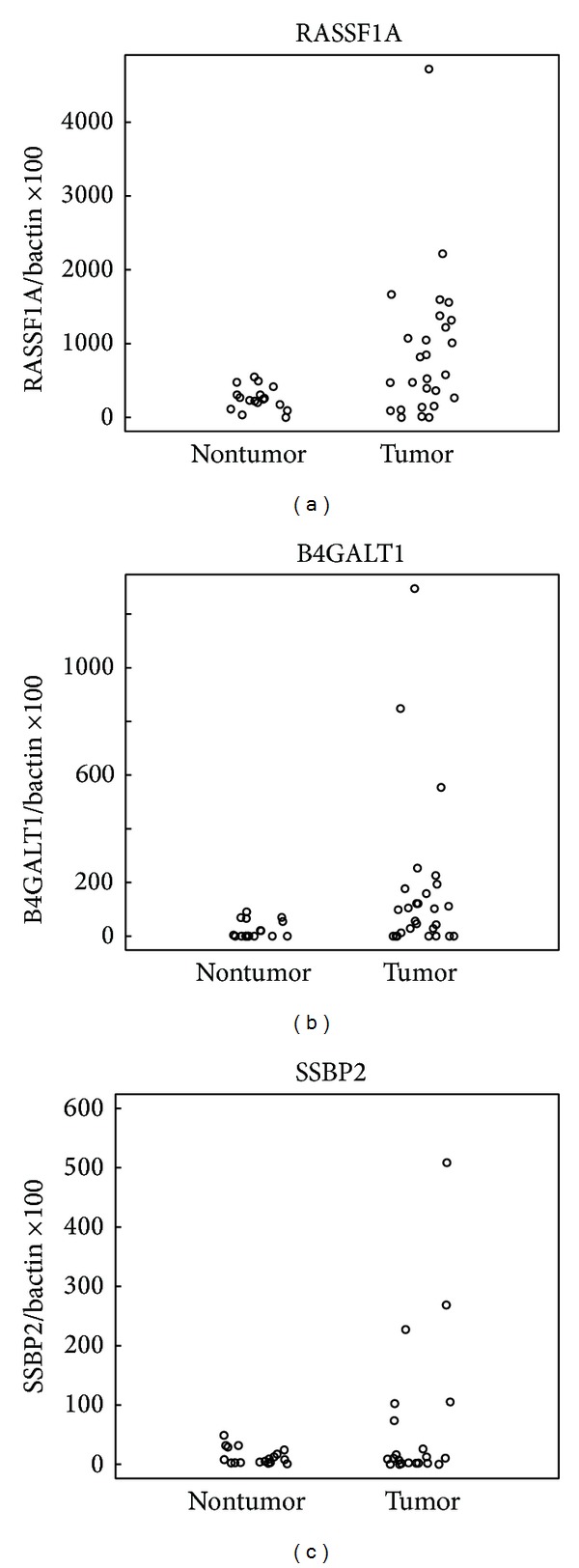
Quantitative MSP results of hepatocellular carcinoma samples and adjacent normal liver samples that were bisulfite treated to examine the promoter methylation status of* RASSF1A*,* B4GALT1*, and* SSBP2*. Scatter plots of quantitative MSP analysis of candidate gene promoters. Twenty-two adjacent normal liver tissue samples and 27 hepatocellular carcinoma samples were tested for methylation for each of the three genes by quantitative MSP. The relative level of methylated DNA for each gene in each sample was determined as a ratio of MSP for the amplified gene to* ACTB* and then multiplied by 100 for easier tabulation (average value of duplicates of gene of interest/average value of duplicates of* ACTB *× 100). The samples were categorized as unmethylated or methylated based on detection of methylation above a threshold set for each gene. This threshold was determined by analyzing the levels and distribution of methylation, if any, in normal, age-matched tissues.

*RASSF1A* was methylated in 14/27 (52%) of HCC samples and in 1/17 (6%) of adjacent normal samples.* B4GALT1* was methylated in 14/27 (52%) of HCC samples and in 0/20 (0%) of adjacent normal samples.* SSBP2* was methylated in 14/27 (52%) of HCC samples and in 6/18 (33%) of adjacent normal samples. Most of the HCC samples (78%) had at least one of these three genes methylated, while less than half of the adjacent normal samples (44%) had one gene methylated. Methylation of at least two of these genes was observed in 70% of the HCC samples and in 0% of the adjacent normal samples (Figures 3(a)-3(b)).

**Figure 4:**
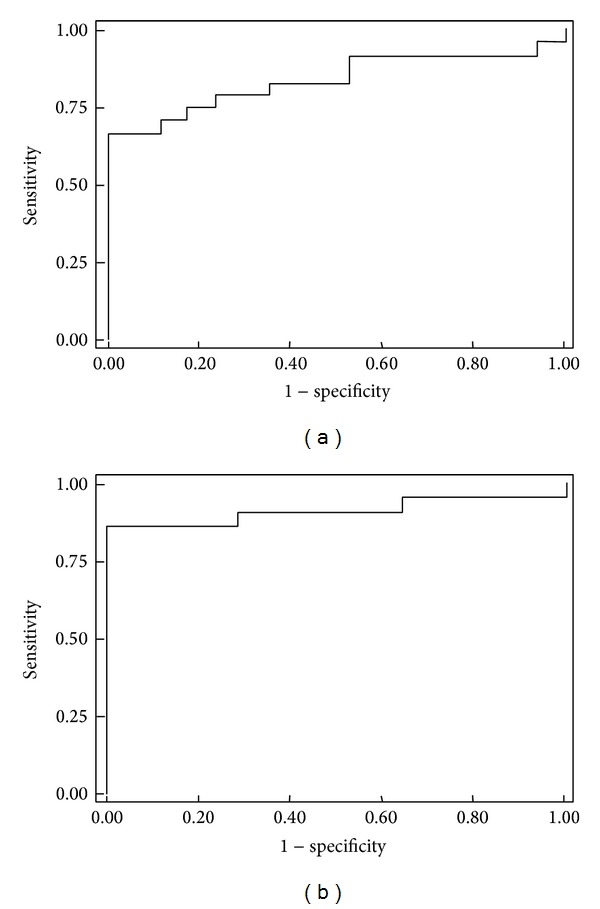
ROC curves for a panel of the three genes* RASSF1A*,* B4GALT1*, and* SSBP2*, individually, (a) and after adjusting a logistic regression model with HCC risk factors: age, gender, ethnicity, and etiology. (b).

ROC curves were used to determine the sensitivity and specificity of the three genes individually and combined in a biomarker panel (Figure 4).* RASSF1A* methylation in the examined tissue samples had a sensitivity of 52%, a specificity of 100%, and an AUC of 0.73 (95% CI, 0.57–0.88).* B4GALT1* methylation exhibited a sensitivity of 52%, a specificity of 100%, and an AUC of 0.75 (95% CI, 0.71–0.89). For* SSBP2*, the sensitivity was 38%, specificity was 100%, and the AUC was 0.58 (95% CI, 0.40–0.75) (Table 2).

When the methylation status of these three genes was included in a logistic regression model together with gender, age, and etiology, the sensitivity was 87%, the specificity 100%, and the AUC was 0.91 (Figure 4(b)). No statistically significant association was observed between patient's clinical data and methylation.

## 4. Discussion

HCC is the most common primary malignancy of the liver in adults, the fifth most common solid tumor, and the third most common cause of cancer death worldwide [22]. HCC incidence and death rates are steadily rising in the United States, and HCC displays the highest average annual percent increase in incidence among the top 15 cancers [23]. HCC patients and people at risk of developing HCC have profound unmet medical and public health needs. Advances in HCC treatment, such as liver transplantation, surgical resection, and loco regional therapies, have only impacted a fraction of the population at risk. More than 70% of HCC patients present with advanced disease and will not benefit from these treatment modalities or from the sole chemotherapeutic agent approved for advanced HCC patients [24].

Epigenetic lesions in DNA without mutations in the coding regions have been shown to be common phenomena in the pathogenesis of a wide range of cancers, especially the methylation-mediated silencing of tumor suppressor genes such as VHL, p16INK4a, E-cadherin, hMLH1, BRCA1, and LKB1 [25, 26]. Moreover, promoter hypermethylation has been linked with a large number of genes involved in HCC including RASSF1A, APC, GSTP1, SOX 17, and RIZ1 [27–29]. Analysis of tissue specimens has revealed that DNA methylation alterations, the best-understood epigenomic biomarker, play a part in a multistage carcinogenetic procedure leading to HCC [27].

Differential methylation has been identified from the early precancerous stages, in association with inflammation and/or persistent infection with HBV or HCV seen in chronic hepatitis or liver cirrhosis to HCC lesions [30]. In addition, concordance of hypermethylation patterns has been shown in matching tissue and plasma DNA from HCC patients [31]. Furthermore, aberrant methylation of a panel of three genes has been reported in serum DNA, 1 to 9 years before clinical diagnosis of HCC [32]. Therefore, unraveling epigenetic alterations in HCC opened up possibilities for discovering new biomarkers for detection and prognosis [9].

By using a study principle that combines promoter-wide and gene-specific methylation platforms that interrogate the promoter region, we were able to distinguish HCC from non-HCC tissue. Our group and others have previously shown that analytical platforms, which quantified global DNA methylation in repetitive regions of the genome, could also distinguish between HCC and non-HCC tissue [33, 34]. DNA methylation alterations in either the promoter or the repetitive elements regions of the genome may therefore serve as useful molecular biomarkers for screening and clinical management for HCC.

The primary goal of our study was to test whether a small sample size is sufficient to provide information on methylation-related studies by using Illumina BeadChip assays and the Nimblegen oligonucleotide arrays. Our discovery set, although including a limited number of samples, was able to identify genes differentially methylated in HCC when compared to normal samples. Among them, there were genes previously reported as also genes with a known role in HCC and other cancer types. To further validate our findings and the power of a genome-wide analysis based on a small sample size, we generated a list of hypermethylated genes in HCC. We ranked the list based on both the frequency in which the genes had been identified in different studies and also in the methylation arrays we used.

*RASSF1A*,* SSBP2*, and* B4GALT1* were selected for further study. Promoter hypermethylation of* RASSF1A*,* SSBP2*, and* B4GALT1* quantified by qMSP had a sensitivity ranging from 38% to 52%, a specificity of 100%, and an AUC from 0.58 to 0.75. A panel combining these genes with HCC risk factors had a sensitivity of 87%, a specificity of 100%, and an AUC of 0.91.

As our knowledge of the HCC epigenome increases, new therapeutic and clinical management strategies may be developed and new serum-based screening or needle biopsy-based diagnostic tools may become available for subgroups at risk for HCC. The pace of DNA methylation translational research is expected to increase exponentially due to the rapid advancement of high-throughput promoter-wide technologies, such as microarray and next-generation sequencing, as well as the advent of user-friendly commercial kits for methylation enrichment [35]. Restoring epigenetically altered pathways is a current research endeavor that will probably lead to the development of new therapeutic tools with translational advantages in malignancies. We are advancing into a new era of individualized molecular medicine, which will allow successful bidirectional interactions between the laboratory bench and patient therapies, based on a better understanding of the genetic and epigenetic mechanisms of human cancers, including HCC.

The aim of this paper was not to provide robust conclusions about specific biomarkers but rather to demonstrate that discrimination between HCC and non-HCC liver tissue using currently available technologies that quantify promoter-wide and gene-specific DNA methylation alterations is feasible. The usefulness of the markers showcased in this paper still needs to be determined in follow-up studies. However, the technological platforms we have used in this project can have an immediate impact on clinical and biomarker development studies.

## 5. Conclusion

Promoter-wide microarray technologies may be used to identify methylation patterns that distinguish between HCC and non-HCC tissue. These technologies are well suited for personalized diagnostics and clinical management. As utilization costs of microarrays decrease, population-based studies may also consider using custom-made microarrays to examine large numbers of participants in prevention and early detection studies. Furthermore, we have also shown how qMSP analyses can be used for fast, accurate, and cost-effective high-throughput validation of methylation frequencies in a large number of samples. There is a potential to test the arising genes' lists to detect biomarkers for early HCC detection in bodily fluids such as plasma, serum, or urine and provide a noninvasive method to clinicians to stratify patients of higher risk for HCC. As the field of translational epigenomics moves forward, clinical tests using these technologies will be warranted to determine their usefulness and reliability in novel screening and clinical management approaches for HCC.

## Supplementary Material

Supplementary Table 1: lists the quantitative Methylation Specific PCR (qMSP) primers and probes used in this manuscript to quantify promoter methylation of *RASSF1A*, *SSBP42*, *B4GALT1* and *ACTB*.

## Figures and Tables

**Table 1:** Hepatocellular carcinoma risk factors per participant.

ID	Age	Sex	Race	Type	Etiology	Histology	Size	AFP	B4GALT1	RASSF1A	SSBP2
16	53	M	W	Normal	Cryptogenic	Cirrhosis	·	·	U	U	U
22	58	M	W	Normal	HCV	Cirrhosis	·	·	U	U	U
30	65	M	W	Normal	HCV	Cirrhosis	·	·	U	U	U
31	67	M	W	Normal	HCV	Cirrhosis	·	·	U	M	M
21	58	M	W	Normal	Other	Cirrhosis	·	·	U	U	U
15	50	F	B	Normal	Cryptogenic	Noncirrhotic	·	·	U	U	U
2	19	F	B	Normal	HCV	Noncirrhotic	·	·	U	U	M
29	62	F	W	Normal	HCV	Noncirrhotic	·	·	U	U	U
9	40	F	B	Normal	Other	Noncirrhotic	·	·	U	U	U
4	26	F	W	Normal	Other	Noncirrhotic	·	·	U	U	M
13	45	F	W	Normal	Other	Noncirrhotic	·	·	U	U	U
35	81	F	W	Normal	Other	Noncirrhotic	·	·	U	M	U
18	54	M	B	Normal	Cryptogenic	Noncirrhotic	·	·	U	U	U
10	42	M	W	Normal	Other	Noncirrhotic	·	·	U	M	U
20	55	M	W	Normal	Other	Noncirrhotic	·	·	U	M	M
36	unk	unk	unk	Normal	unk	unk	·	·	U	U	M
37	unk	unk	unk	Normal	unk	unk	·	·	U	U	U
38	unk	unk	unk	Normal	unk	unk	·	·	U	U	M
39	53	M	M	Normal	ETOH	Noncirrhotic	·	·	U	U	U
40	19	F	F	Normal	HCV	Noncirrhotic	·	·	U	U	U
41	57	M	M	Normal	HCV	Cirrhosis	·	·	U	U	U
42	60	M	M	Normal	HCV	Cirrhosis	·	·	U	U	U
5	28	F	W	Tumor	Other	HCC	2.5	1	M	U	U
28	62	F	W	Tumor	HCV	HCC	2.8	17	U	M	M
17	53	M	W	Tumor	Cryptogenic	HCC	4	10	U	M	U
7	40	F	B	Tumor	Other	HCC	3.7	1	U	U	M
25	59	F	AS	Tumor	HBV	HCC	4	20	M	M	M
3	20	F	W	Tumor	HCV	HCC	4	unk	M	U	U
27	60	M	B	Tumor	HCV/ETOH	HCC	4	20	M	M	M
11	45	M	W	Tumor	HCV	HCC	5.0	unk	M	M	M
8	40	M	W	Tumor	HCV	HCC	5.5	11110	U	U	U
32	67	M	W	Tumor	HCV	HCC	6	2	M	M	M
33	73	M	W	Tumor	Other	HCC	6	2	U	M	M
6	37	M	B	Tumor	Other	HCC	7.0	54071	U	M	U
23	58	M	W	Tumor	Other	HCC	7.2	4659	M	U	M
14	50	F	B	Tumor	Cryptogenic	HCC	9	1594	U	M	U
26	60	M	W	Tumor	Cryptogenic	HCC	12	146	M	M	M
19	55	M	W	Tumor	Other	HCC	17	5	U	M	M
12	45	F	B	Tumor	HVB/HCV	HCC	unk	unk	U	U	U
24	58	M	W	Tumor	HCV	HCC	unk	unk	M	M	M
1	19	F	B	Tumor	HCV	HCC	25	19764	U	M	M
34	74	F	W	Tumor	Cryptogenic	HCC	4.5	unk	M	M	U
43	42	M	M	Tumor	Other	HCC	15	NA	M	M	U
44	26	F	M	Tumor	Other	HCC	8	NA	M	M	U
45	12	F	M	Tumor	Other	HCC	NA	NA	M	M	U
46	NA	NA	NA	Tumor	NA	HCC	NA	NA	U	M	U
47	NA	NA	NA	Tumor	NA	HCC	NA	NA	U	M	M
48	NA	NA	NA	Tumor	NA	HCC	NA	NA	U	U	M
49	45	F	F	Tumor	Cryptogenic	HCC	1.5	NA	M	M	M

M: Methylated; U: Unmethylated; unk: unknown.

**Table 2:** Specificity, sensitivity, and area under the curve results for RASSF1A, B4GALT1, and SSBP2 in HCC, individually, and in a combined panel of the three genes.

	RASSF1A	B4GALT1	SSBP2	Combined panel of 3 genes
Specificity	100%	100%	100%	100%
Sensitivity	52%	52%	38%	68%
AUC	0.73	0.75	0.58	0.82
