# Supplementary material for: Genome-Wide and Gene-Specific Epigenomic Platforms for Hepatocellular Carcinoma Biomarker Development Trials
Source: Gastroenterol Res Pract. 2014 Apr 17;2014:597164. doi: 10.1155/2014/597164 (PMC4009191; doi:10.1155/2014/597164)
Supplement: Supplementary file 1 — Supplementary Table 1: lists the quantitative Methylation Specific PCR (qMSP) primers and probes used in this manuscript to quantify promoter methylation of RASSF1A, SSBP42, B4GALT1 and ACTB. [file 597164.f1.pdf]

Supplementary Table 1. QMSP Primer and probe information

Gene	Forward 5'-3'	Reverse 5" – 3"	Genbank #	Annealing Temperature
**ACTB**	TGG TGA TGG AGG AGG TTT AGT AAG T	AAC CAA TAA AAC CTA CTC CTC CCT TAA	Y00474	60
**RASSF1A**	GCGTTGAAGTCGGGGTTC	CCCGTACTTCGCTAACTTTAAACG	NM_007182	60
**B4GALT1**	TAGGAAACGGGTTTCGACG	CCGTCCACTTTCTTTACCG	NM_001497	58
**SSBP2**	ATTTTTGCGGTCGTAGCGGT	TTCTACGACAAATCTAACGAA	NM_012446	60
	**Probe 6FAM 5´-3´TAMRA**	**Amplicon size (Nucleotide range)**		
**ACTB**	ACC ACC ACC CAA CAC ACA ATA ACA AAC ACA	133 bp; (390–522)		
**RASSF1A**	ACAAACGCGAACCGAACGAAACCA	75 bp; (45-119)		
**B4GALT1**	CGTTAAACAACGAAATCCAACCGAA	116 bp; (583-688)		
**SSBP2**	ATATCCAAAACGCCGCGAAACTCC	89bp; (694-784)		

Supplementary Material:

Supplementary Table 1 lists the quantitative Methylation Specific PCR (qMSP) primers and probes used in this manuscript to quantify promoter methylation of *RASSF1A, SSBP42, B4GALT1* and *ACTB.*

We use a two-step process to design the qMSP primers and probes used to validate with PCR the candidate genes identified with methylation arrays:

Step 1 – Bisulfite sequencing primer design and evaluation

Step 2 – qMSP primers and probes design and evaluation

Step 1- Bisulfite sequencing primer design and evaluation

1. Look for the target gene in UCSC Genome Browser
2. Choose the target gene sequence listed under RefSeq Genes
3. Click the link to Genome Sequence from assembly
4. In the new window called "Get Genomic Sequence Near Gene" mark the following options:
    a. Promoter/1200 bases upstream from TSS
    b. 5' UTR exons
    c. Downstream 600 bases from TSS
    d. One FASTA record per gene.
    e. Exons in upper case, everything else in lower case.
5. Click in submit and copy the genomic sequence
6. In a new window open MethPrimer (http://www.urogene.org/methprimer/index1.html)
7. Paste the genomic sequence into the MethPrimer window.
8. Click on "Pick primers for bisulfite sequencing PCR or restriction PCR".
9. Select the primer pair of your choice (MethPrimer provides five choices) and copy them to Word, together with the bisulfite-converted sequence they will amplify (you need to erase the upper sequence after you perform the copy past, as the upper sequence is the genomic sequence).
10. Find the primers in the sequence and highlight them.
11. Order the primers from you oligonucleotide provider of choice.
12. Perform touchdown PCR and send for Sanger sequencing to corroborate if the primers are amplifying the genomic fragment of interest.

Step 2

1. Paste the genomic sequence selected in Step 1.4 into a new window of MethPrimer (http://www.urogene.org/methprimer/index1.html)
2. Click on "Pick MSP Primers"
3. A new window appears with the results of several methylated and unmethylated primer sets. To Choose the adequate primers you must consider:
    a. primer pairs that span CpGs islands.
    b. primer pairs that have a CG in the 3' end
    c. an amplicon size of 120-200 bp
4. Save genomic sequence and MethPrimer results in a Word file.

5. Order the primers from your oligonucleotide provider of choice.
6. Validate the methylated and unmethylated primers on normal and cancer cell line DNA.
7. Use Sanger sequencing to troubleshoot and/or improve qMSP probe design.
8. After the qMSP primers are validated design the qMSP probe using the fragment amplified by the selected primers (manual design)
    a. The annealing temperature must be 10°C higher than primers.
    b. The size must be between 15-25 bp
    c. Sometimes the qMSP works better if you choose an antisense probe
9. Order the Probe from your oligonucleotide vendor of choice
